# Management of acute cholecystitis in patient with cyclic neutropenia: a case report

**DOI:** 10.1186/s40792-021-01117-7

**Published:** 2021-01-23

**Authors:** Saki Nishikawa, Michinori Hamaoka, Hideki Nakahara, Toshiyuki Itamoto

**Affiliations:** 1 Department of Surgery, Hiroshima Prefectural Hospital, 1-5-54 Ujina-Kanda, Minami-ku, Hiroshima, 734-8530 Japan; 2 Department of Pathology, Hiroshima Prefectural Hospital, 1-5-54 Ujina-Kanda, Minami-ku, Hiroshima, 734-8530 Japan

**Keywords:** Acute cholecystitis, Cyclic neutropenia, Laparoscopic cholecystectomy, Operation, Surgery

## Abstract

**Background:**

Cyclic neutropenia is a disease that causes a neutropenic decrease in peripheral blood in a cycle of about 21 days. It is a rare hereditary disorder with an estimated incidence of 0.5–1 cases per million population. The absolute neutrophil count can drop to zero, and neutropenic nadir may last for 3–5 days. This is a rare disease, and there are few reports of abdominal surgery in cyclic neutropenia patients; thus, we report this case of neutrophil count fluctuation and perioperative management.

**Case presentation:**

A 31-year-old man with cyclic neutropenia was transferred to our hospital complaining of right season rib pain, but no rebound tenderness. His C-reactive protein was elevated (4.37 mg/L) and computed tomography revealed a large number of small stones in the gallbladder body and an incarceration in the gallbladder neck. He was diagnosed with acute cholecystitis. Ideally, surgical intervention should have been performed immediately, but because his neutrophil count was 300/μL, endoscopic naso-gallbladder drainage was performed and he was provided antibiotics until his neutrophil count increased to acceptable levels. Three days after admission, his neutrophil count had increased and laparoscopic cholecystectomy was performed. For one week after the operation, antibiotics were administered; he had an uneventful postoperative recovery. He was discharged on the seventh postoperative day and provided an oral antibiotic.

**Conclusions:**

Infection can be serious in patients with cyclic neutropenia, and it is therefore, important to determine the timing of surgery and to apply appropriate perioperative management with drainage and antibiotic administration.

## Background

Cyclic neutropenia is a disease that causes a neutropenic decrease in peripheral blood in a cycle of about 21 days. It is a rare hereditary disorder with an estimated incidence of 0.5–1 cases per million population and there is no gender difference in the incidence [1]. Familial cases of autosomal dominant inheritance have been reported, and it may be caused by the neutrophil elastase gene (ELA2) mutation. The absolute neutrophil count can drop to zero, and neutropenic nadir may last for 3 to 5 days [2]. The most common infections are pharyngitis, gingivitis, and periodontitis during neutropenic nadir, but serious symptoms can occur during neutropenia [1]. Perioperative complications can be fatal for these patients; hence, perioperative management requires particular attention. Moreover, though acute cholecystitis is a common disease of the abdomen, its clinical manifestation can be severe. The symptoms may be more severe during neutropenia in these patients; therefore, it is important to determine the timing of surgery considering the change in absolute neutrophil count and perform more careful perioperative management.

## Case presentation

**Fig. 1:**
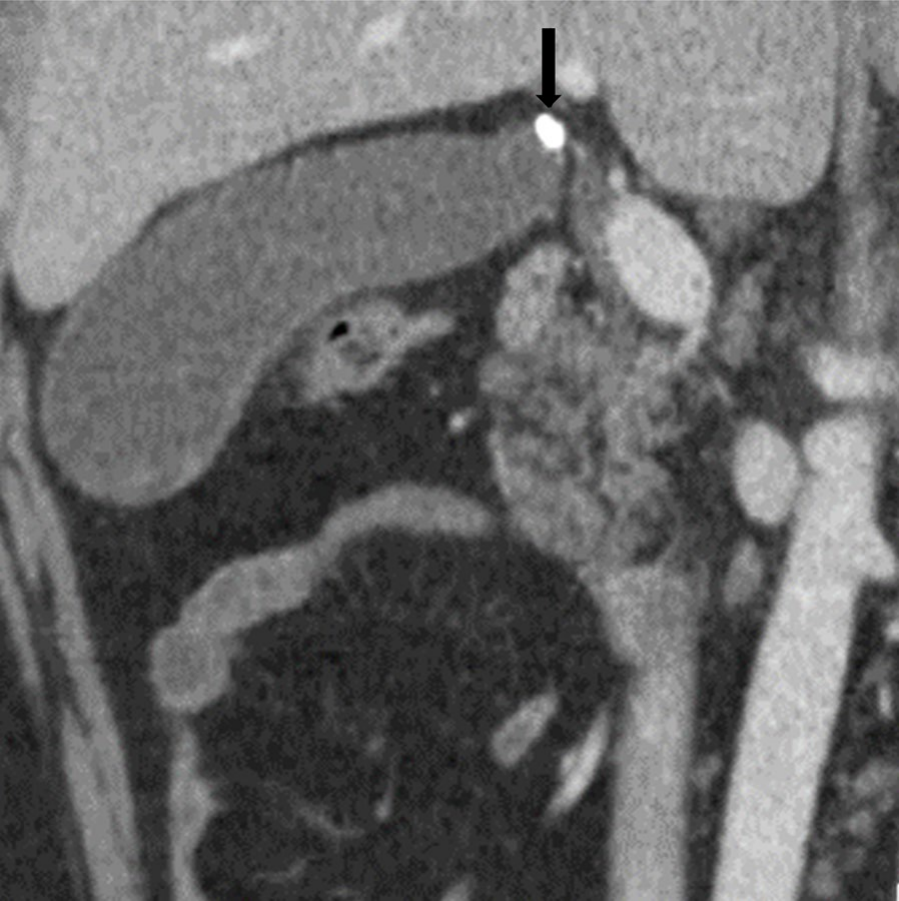
Computed tomography. Arrow indicates incarceration of a small in the neck of the gallbladder

**Fig. 2:**
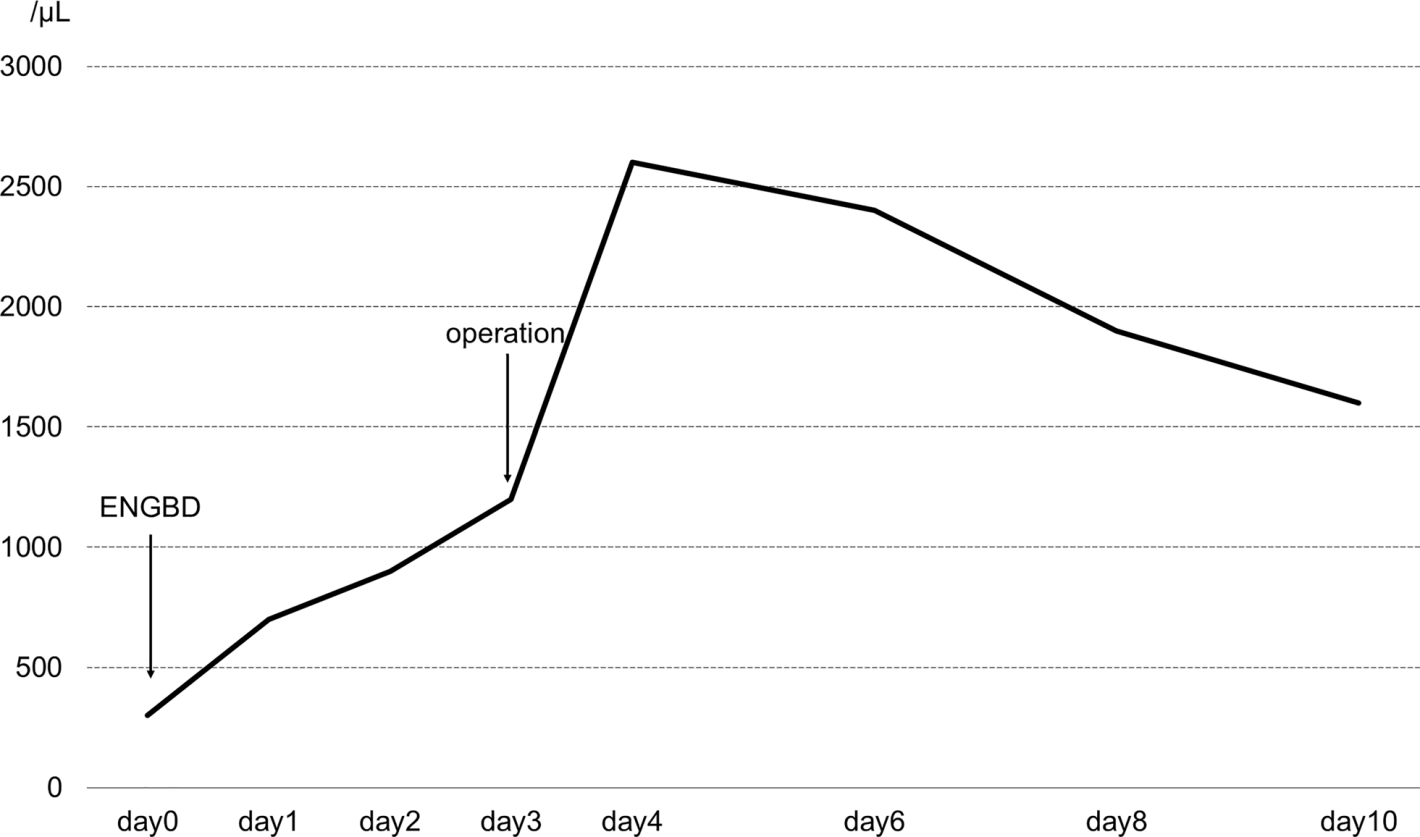
Changes in the number of neutrophils. The vertical axis represents the number of neutrophils, the horizontal axis represents the number of days after hospitalization, and the polygonal line represents the fluctuation in the number of neutrophils

A 31-year-old man with cyclic neutropenia was transferred to our hospital complaining of right side rib pain. He was diagnosed with the disease in early childhood and his neutrophil count varied from 0 to 40% (0–3000/μL) in a cycle of approximately 21 days. During neutropenic nadir, he had a fever and aphthous stomatitis, but these symptoms improved in a few days. When his symptoms were severe, he was occasionally prescribed antibiotics and granulocyte colony-stimulating factor (G-CSF). He presented with pain from the epigastric region to the right season rib, but no recoil. His C-reactive protein (CRP) was elevated (4.37 mg/L) and computed tomography (CT) revealed a large number of small stones in the gallbladder body and an incarceration in the gallbladder neck (Fig. 1). He was diagnosed with acute cholecystitis. His white blood cell count was 3300/μL and neutrophil count was 300/μL. Endoscopic naso-gallbladder drainage (ENGBD) was performed, and antibiotics were administered. Three days after admission, his neutrophil count increased (Fig. 2), and laparoscopic cholecystectomy was performed. For 1 week after the operation, antibiotics were instilled, and he had an uneventful postoperative recovery; this was changed to an oral antibiotic on his discharge on the 7th day after the operation.

## Discussion

Cyclic neutropenia is a rare autosomal dominantly inherited disorder. It is characterized by periodic neutropenia that recurs every 14–35 days, although most patients exhibit a cycle of about 21 days. The disorder most frequently starts in infancy or childhood. Most often, it is benign, and the symptoms ameliorate as the patient grows older [3]. The most common symptoms are fever, pharyngitis, gingivitis, and periodontitis. The patients may have more severe symptoms at the nadir of neutropenia, and infectious deaths have been reported in 10% of patients [3]. When surgery is required for these patients, possible worsening of the condition and severe postoperative complications should be considered due to neutropenia. Table1 shows data for abdominal surgeries performed in emergency settings for patients with cyclic neutropenia acquired via searching PubMed using the terms “cyclic neutropenia”, and “operation” [1, 4–6]. In these cases, the absolute neutrophil count was as low as 0–200/μL at the first visit. Patients who received G-CSF were discharged without postoperative complications, but patients who did not receive G-CSF experienced complications such as enterocutaneous fistula, intestinal necrosis, and intra-abdominal abscess. Patients who experienced complications underwent reoperation at the time of neutrophil elevation and experienced positive outcomes moving forward. However, these postoperative complications can lead to potentially fatal peritonitis and sepsis. Neutrophil count is considered to be significantly involved in the postoperative course. Therefore, it is important to determine the timing of the surgery and prevent postoperative infections while taking into consideration the patient's general condition and absolute neutrophil count.

**Table 1:** Summary of abdominal surgeries performed in emergency settings for patients with cyclic neutropenia

Author/year	Age/sex	Disease	Neutrophil count at the first visit	Use of G-CSF	Prognosis
Geelhoed et al./1973 [4]	10/male	Neutropenic enterocolitis	0	No	Survival
Langer et al./1990 [6]	4/male	Neutropenic enterocolitis	0	No	Survival
O'Hanrahan et al./1991 [5]	14/female	Neutropenic enterocolitis	200	No	Survival
Nedeljka Glavan et al./2015 [1]	4/male	Acute appendicitis	0	Yes	Survival
Saki Nishikawa et al./2020	31/male	Acute cholecystitis	300	No	Survival

As a way of raising neutrophil count, recombinant G-CSF has proved efficacious. However, there are no universally accepted guidelines regarding the dose and duration of G-CSF treatment as well as no guidelines regarding the timing of surgery in the disorder.

Neutrophil count typically well exceeds 1500/μL, once the neutrophil count is below 1000/μL, the patient becomes susceptible to infections [2]. Patients respond to G-CSF doses in the range of 2–3 μg/kg, administered subcutaneously either daily or on alternate days [7].

Our patient underwent ENGBD and antibiotics were administered. Gallbladder drainage is recommended as a useful treatment for patients with acute cholecystitis who are at high risk for surgery and who cannot be operated on immediately for reasons stipulated in the 2018 Tokyo Guidelines [8]. Inferred from the patient's neutropenia cycle, G-CSF was not provided before ENGBD because the neutropenic nadir had passed and the neutrophil count was relatively well maintained at 300/μL. After ENGBD insertion, his general condition was stable and neutrophil count rose without the administration of G-CSF. Laparoscopic cholecystectomy was performed when his absolute neutrophil count was above 1000/μL.

## Conclusion

Determination of appropriate timing of surgery enables safe surgery for patients with neutropenia. Appropriate antibiotics and gallbladder drainage proved effective until neutrophil levels were sufficiently elevated.

## Data Availability

Data supporting the conclusions are included in the article.

## Abbreviations

ELA2: Elastase gene
CRP: C-reactive protein
CT: Computed tomography
ENGBD: Endoscopic naso-gallbladder drainage
G-CSF: Granulocyte colony-stimulating factor
